# Low ankle–brachial index is associated with higher cardiovascular mortality in individuals with nonalcoholic fatty liver disease

**DOI:** 10.1186/s40001-024-01878-5

**Published:** 2024-05-09

**Authors:** Guang Xiong, Liuqing Guo, Liwei Li, Min Liang

**Affiliations:** 1 Department of Geriatric Endocrinology and Metabolism, The First Affiliated Hospital of Guangxi Medical University, No. 6 Shuangyong Road, Nanning, 530021 Guangxi China; 2 Department of Gastroenterology, The Second Affiliated Hospital of Guangxi Medical University, Nanning, China

**Keywords:** Nonalcoholic fatty liver disease, Ankle–brachial index, Mortality

## Abstract

**Background and aims:**

Ankle brachial index (ABI) is a risk factor for cardiovascular mortality, but it is unclear whether ABI is associated with cardiovascular mortality in patients with nonalcoholic fatty liver disease (NAFLD). The current study aimed to evaluate the association between ABI with cardiovascular and all-cause mortality in patients with NAFLD.

**Methods:**

We performed a cohort study using the data of the1999–2004 National Health and Nutrition Examination Survey data of adults. Mortality data were followed up to December 2015. NAFLD was defined by the hepatic steatosis index or the US fatty liver index. ABI was classified into three groups: ABI ≤ 0.9 (low value); 0.9 < ABI ≤ 1.1 (borderline value); ABI greater than 1.1 (normal value).

**Results:**

We found that low ABI was associated with an increased risk of cardiovascular mortality in patients with NAFLD (HR: 2.42, 95% CI 1.10–5.33 for low value ABI vs normal value ABI, P for trend = 0.04), and the relationship was linearly and negatively correlated in the range of ABI < 1.4. However, low ABI was not associated with all-cause mortality in patients with NAFLD. Stratified by cardiovascular disease, ABI remains inversely correlated with cardiovascular mortality in NAFLD patients without cardiovascular disease. Stratified by diabetes, ABI is inversely correlated with cardiovascular mortality in NAFLD patients regardless of diabetes status.

**Conclusions:**

Low ABI is independently associated with higher cardiovascular mortality in NAFLD cases. This correlation remains significant even in the absence of pre-existing cardiovascular disease or diabetes.

**Supplementary Information:**

The online version contains supplementary material available at 10.1186/s40001-024-01878-5.

## Introduction

Non-alcoholic fatty liver disease (NAFLD) has become the most common chronic liver disease globally, paralleling the rise in global obesity and Type 2 diabetes mellitus [1, 2]. It is estimated that its prevalence is around 25% worldwide, gradually becoming a significant public health issue [1]. NAFLD is closely associated with metabolic disorders, and cardiovascular mortality (CVM) has been identified as the most common cause of death among NAFLD patients [3–6].

The ankle–brachial index (ABI) is a simple, non-invasive measurement method, calculated as the systolic blood pressure (SBP) ratio of the ankle artery to the brachial artery [7]. ABI is also a risk factor for arteriosclerosis and cardiovascular mortality, associated with increased CVM in populations with CVD, diabetes, and renal insufficiency [8–10]. However, the specific relationship between ABI and mortality in NAFLD patients has not been fully explored. Given the high metabolic burden and increased cardiovascular risk in NAFLD patients, ABI appears to be a valuable prognostic tool for this group. Identifying low ABI in NAFLD patients could help stratify risk, guide management strategies, and potentially intervene early to reduce mortality risk. Given its non-invasive nature and ease of measurement, ABI can be measured quickly by properly trained professionals within primary healthcare facilities [7]. With a high prevalence of NAFLD and the majority of NAFLD patients receiving care in primary health settings [11], routine ABI measurements is likely to offer significant benefits for individuals with NAFLD.

Therefore, this study aims to explore the predictive value of ABI for all-cause mortality and cardiovascular mortality in NAFLD patients. We seek to elucidate the utility of ABI in predicting mortality risk among NAFLD patients and its potential role in improving patient prognosis through better risk stratification.

## Methods

### Study sample

The participants in this study were recruited from the NHANES which is a non-institutionalized stratified probability sampling survey [12]. Because the ankle–brachial index was only studied from 1999 to 2004, we combined data from 1999 to 2004 to create a sample of 31,126 subjects. Of them, 15,332 were over the age of 20. We also excluded 8928 subjects who met the following criteria: (1) excessive drinking (> 14 and > 21 standard drinks weekly in women and men, respectively) (*n* = 362); (2) seropositive for hepatitis B or C virus (*n* = 293); (3) taking medications that can affect hepatic steatosis (*n* = 495); (4) pregnant women (*n* = 822); (5) missing ABI data (*n* = 6499); (6) ABI > 1.4 (high ABI, not the focus of our study) (*n* = 43); (7) missing the information required to define NAFLD by hepatic steatosis index (HSI, details were described below) (*n* = 414). After excluding the aforementioned population, there were 6404 people left, 3649 of whom were classified as having NAFLD (HSI > 36) and had their ABI measured.

**Fig. 1:**
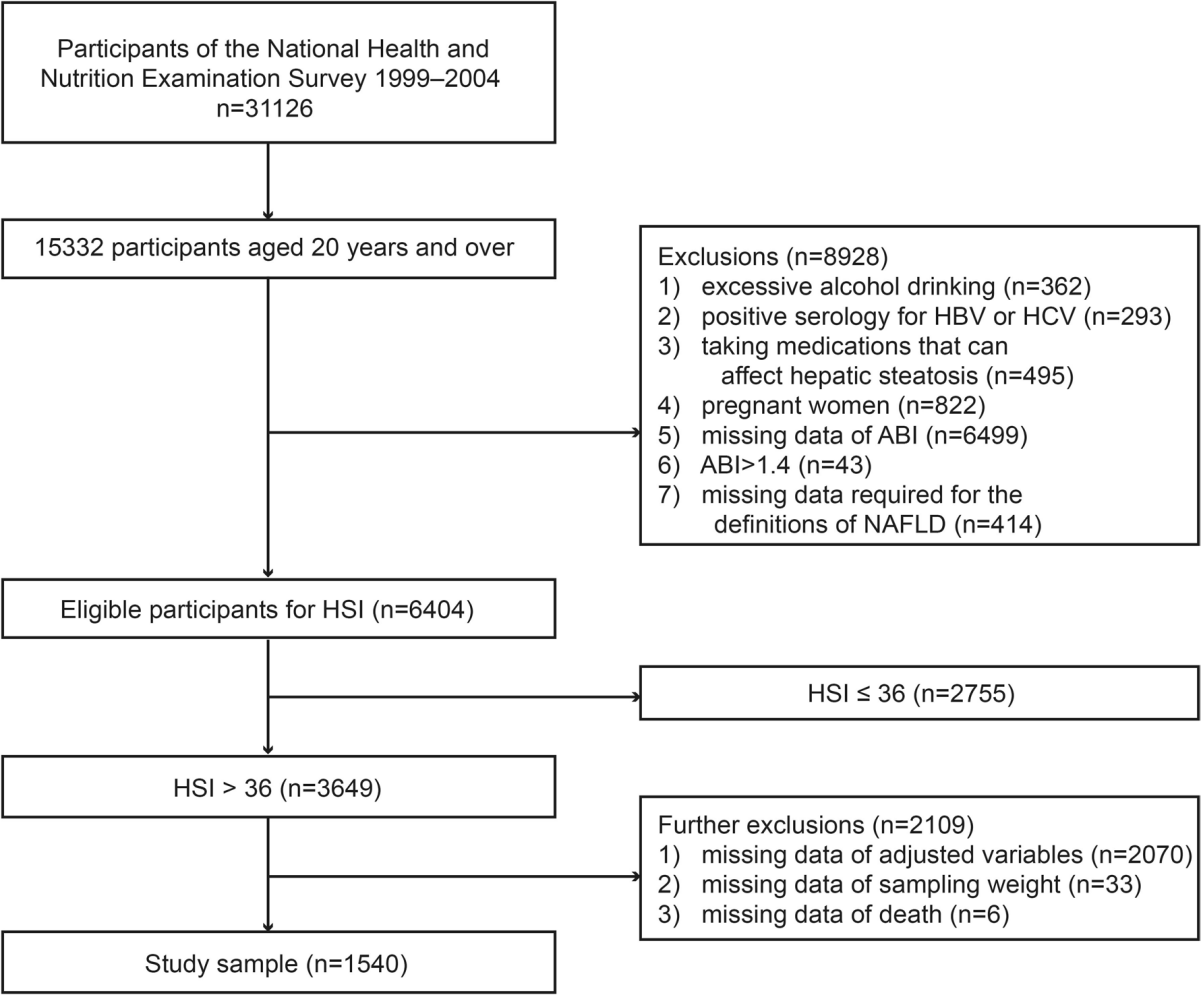
Flow diagram of participants defined by hepatic steatosis index in the study

Due to incomplete covariate data, we further excluded 2070 individuals. This group comprised those with missing waist circumference (*n* = 30), missing high density lipoprotein-cholesterol (HDL-cholesterol) (*n* = 2), missing low density lipoprotein-cholesterol (LDL-cholesterol) (*n* = 2030), missing triglycerides (*n* = 2), missing fasting blood glucose (*n* = 3), and missing fasting insulin (*n* = 3). Considering the potential bias introduced by excluding these participants, we conducted a comparison of baseline characteristics between the population before and after exclusion (Table 1). The results indicated that the majority of variables showed no significant differences, particularly LDL levels, which remained consistent across both groups. Additionally, we excluded 33 subjects due to missing sampling weight data and 6 subjects due to missing mortality data. The total number of participants with NAFLD defined by HSI was 1540 (Fig. 1). We used the same method to screen the study sample of NAFLD diagnosed based on the US fatty liver index (USFLI, details were described below) for sensitivity analysis, and the sample of USFLI eventually included 1026 individuals (Supplementary Fig. 1).

**Table 1:** Characteristics of before and after the exclusion of covariates

	Pre-exclusion (*n* = 3649)	Post-exclusion (*n* = 1540)	*P*-value
Ankle–brachial index	1.09 ± 0.13	1.09 ± 0.14	0.92
Age (years)	58.40 ± 11.6	58.49 ± 11.91	0.81
Gender (%)			
Male	717 (46.6)	1741 (47.7)	0.47
Female	823 (53.4)	1908 (52.3)	
Ethnicity (%)			
Non-Hispanic white	774 (50.3)	1830 (50.2)	0.89
Non-Hispanic black	278 (18.1)	684 (18.7)	
Mexican American	390 (25.3)	895 (24.5)	
Others	98 (6.4)	240 (6.6)	
Education level (%)			
Less than high school	519 (33.7)	1248 (34.2)	0.90
High school or equivalent	362 (23.5)	865 (23.7)	
College or above	659 (42.8)	1536 (42.1)	
Marital status (%)			
Married or living with partner	1025 (66.6)	2405 (65.9)	0.68
Others	515 (33.4)	1244 (34.1)	
Family income-to-poverty ratio (%)			
≤ 1.0	196 (12.7)	500 (13.7)	0.38
> 1.0	1228 (79.7)	2847 (78.0)	
Unknown	116 (7.5)	302 (8.3)	
Smoker (%)			
Never	751 (48.8)	1795 (49.2)	0.92
Ever	547 (35.5)	1275 (34.9)	
Current	242 (15.7)	579 (15.9)	
BMI (kg/m^2^)	32.00 ± 4.85	31.92 ± 4.87	0.63
Waist circumference (cm)	106.53 ± 11.76	106.55 ± 11.88	0.94
Hypertension (%)			
Without	634 (41.2)	1525 (41.8)	0.70
With	906 (58.8)	2124 (58.2)	
Diabetes (%)			
Without	1203 (78.1)	2723 (74.6)	0.01
With	337 (21.9)	926 (25.4)	
CVD (%)			
Without	1325 (86.0)	3097 (84.9)	0.30
With	215 (14.0)	552 (15.1)	
PA level (%)			
Low	707 (45.9)	1746 (47.8)	0.44
Moderate	325 (21.1)	749 (20.5)	
High	508 (33.0)	1154 (31.6)	
HDL-cholesterol (mg/dL)	49.95 ± 13.66	48.95 ± 13.68	0.02
LDL-cholesterol (mg/dL)	128.15 ± 35.64	127.82 ± 35.75	0.80
Triglyceride (mg/dL)	153.03 ± 71.3	181.98 ± 186.26	< 0.01
Fasting glucose (mg/dL)	111.51 ± 33.76	116.66 ± 44.26	< 0.01
Fasting insulin (pmol/L)	87.39 ± 58.22	100.93 ± 104.45	< 0.01

Data in the table: continuous variables are expressed as the weighted mean ± standard error; categorical variables are expressed as unweighted frequencies (weighted  percentages)

BMI: body mass index; CVD: cardiovascular disease; PA: physical activity; HDL: high density lipoprotein; LDL: low density lipoprotein

### Measurement of ABI

ABI measurements were taken while the subjects were supine (parks Mini lab IV, model 3100) [13]. The subjects' SBP was measured in the ankle (posterior tibial artery) and right arm (brachial artery) in this study [13]. We measured SBP in the left arm if the subject had any condition on the right arm that could not be measured or might affect the precision of the measurement [13]. Subjects under the age of 60 were measured twice at each site, whereas subjects over the age of 60 were measured once at each site [13]. The mean SBP in bilateral ankles was then divided by the mean arm SBP to obtain left and right ABI separately (values of subjects aged 60 and over were calculated by the one-time value) [13]. The lower one of the left and right ABI values was then taken as the individual ABI [13].

### Definition of NAFLD

For individual patients, especially in tertiary care settings, imaging studies are preferred, but for larger-scale research, serum biomarkers are favored due to the feasibility issues related to the availability and cost of imaging [14, 15]. NHANES is a large-scale population health survey that lacks liver ultrasonography for steatosis in most of its cycles. Therefore, we opted for non-invasive models based on serological indicators to define NAFLD. For sensitivity analysis, we used two non-invasive methods to define NAFLD: the HSI (hepatic steatosis index) and the USFLI (US fatty liver index) [16, 17]. NAFLD is defined as having HSI > 36 and USFLI ≥ 30, respectively. Both methods were obtained and validated in a non-institutionalized large-scale population health examination, and they all performed well in terms of diagnostic performance [16, 17]. The HSI, with an area under the receiver-operating curve of 0.812, diagnoses NAFLD at values > 36 and excludes it at < 30, with sensitivities and specificities of 93.1% and 92.4%, respectively [16]. We used these two methods to diagnose NAFLD after excluding subjects with other chronic liver diseases (details have been described above) [18].

HSI = 8 × (alanine aminotransferase)/(aspartate aminotransferase) + BMI (+ 2,if DM; + 2,if female),

USFLI = (e^−0.8073*non−Hispanic black+0.3458*Mexican American+0.0093*age+0.6151*loge(gamma−glutamyl transferase) +0.0249*waist circumference+1.1792*loge(insulin) +8242*loge(glucose) −14.7812^) / (1 + e^−0.8073*non−Hispanic black+0.3458*Mexican American+0.0093*age+0.6151*loge(gamma−glutamyl transferase) +0.0249*waist circumference+1.1792*loge(insulin) +8242*loge(glucose) −14.7812^) * 100.

### Covariates

NHANES provided us with sociodemographic information, examination data, laboratory data, and questionnaire data [12]. Covariates were selected based on risk factors identified in previous studies [19, 20]. Additionally, research focusing on risk factors for mortality among NAFLD patients was also consulted [21]. The preliminary covariates to be included are: age, gender, ethnicity, education level, marital status, economic status, body mass index (BMI), waist circumference, hypertension, diabetes, cardiovascular disease, and physical activity. Additionally, total cholesterol (TC), HDL-cholesterol, LDL-cholesterol, triglycerides, fasting blood glucose, and fasting insulin levels are also considered. There were three levels of education: high school and below, high school and equal education, and college and above. Non-Hispanic White, Mexican American, non-Hispanic Black and others were the race categories. Meanwhile, this work classified marital status as married/living with a partner and other status. According to the ratio of family income to poverty guideline, the economic situation was classified as ≤ 1.0, > 1.0, or unknown. Smoking habits were divided into three categories: never smokers (smoking < 100 cigarettes in their lifetime), current smokers (still smoking, > 100 cigarettes in their lifetime), and ex-smokers (ever smoking, > 100 cigarettes in their lifetime). Diabetes was defined as subjects who met at least one of the criteria listed below [22]. The criteria were as follows: (1) a diagnosis of diabetes; (2) use of diabetes medications or insulin; (3) fasting blood glucose ≥ 126 mg/dL; and (4) hemoglobin A1c ≥ 6.5%. Hypertension was defined as meeting one or more of the following criteria [23]: (1) a history of hypertension; (2) SBP ≥ 140 mmHg and/or diastolic blood pressure (DBP) ≥ 90 mmHg; and (3) use of blood pressure medication. Cardiovascular disease (CVD) is defined as being informed by a health professional or a physician of the following conditions: coronary heart disease, congestive heart failure, heart attack, angina/angina pectoris, and stroke. Furthermore, this study divided physical activity into three levels based on the metabolic equivalent task level (MET-min: metabolic equivalent-minute) of moderate-to-vigorous physical activity (MVPA) per week [24, 25], which were as follows: (1) low (< 600 MET-min MVPA/week); (2) moderate (600–1500 MET-min MVPA /week); and (3) high (> 1500 MET-min MVPA /week).

### Mortality

Each NHANES subject's death status was linked with NDI (National Death Index) death data, and they were all followed up on until December 31, 2015 [26]. ICD-10 was used to determine the cause of death: UCOD_113 (underlying cause of death 113) codes 55–64 and 70 are attributed to CVD [26].

### Statistical analysis

Since the complex sampling survey design was used in the NHANES, this work used appropriate stratification, clustering, and sample weight to reflect the overall situation of the population in accordance with the NHANES analysis guidelines [27]. The baseline data were presented as the weighted mean ± standard error or weighted frequency (95% confidence interval). To compare the differences of continuous variables, the Kruskal–Wallis test or one-way ANOVA was used, and the Chi-square test was used to compare the differences between classified variables. Considering the multitude of covariates, we conducted collinearity analysis and removed certain covariates to mitigate the risk of overadjustment in the constructing the survival analysis model and to enhance the study's power. Given that NAFLD was defined using the HSI and USFLI, adjusting for the components within these two formulas could increase collinearity. Consequently, we removed specific components for each model (for the model based on HSI, BMI was removed; for the model based on USFLI, ethnicity, age, waist circumference, blood glucose, and fasting insulin were excluded). Furthermore, by calculating the Variance Inflation Factor values within the model, we identified and cautiously reduced covariates causing collinearity, which led to the further elimination of TC. Based on the criteria listed below, this study classified ABI into three groups [7]: (1) ABI ≤ 0.9 (low value); (2) 0.9 < ABI ≤ 1.1 (borderline value); (3) ABI greater than 1.1 (normal value). ABI was included in the regression model analysis as a continuous and a categorical variable, respectively, and we used multivariate Cox regression to investigate the relationship between ABI and mortality. When ABI is converted into a categorical variable, we enter the model with the median value of ABI in each group as a continuous variable to test the linear trend. In the fully adjusted model, the linear relationship between ABI and mortality was also evaluated using restricted cubic spline functions of three knots (the 5th, 50th, and 95th percentiles of ABI). This study further examined the relationship between ABI and mortality stratified by with and without cardiovascular disease, and the interactions of CVD and ABI were tested. In this study, all tests were two-sided, with a difference of *P* < 0.05 indicating significance. All statistical analyses were conducted using R 4.2.3 (http://www.R-project.org; The R Foundation).

## Results

In total, 1540 people took part in this study. The baseline patient characteristics are depicted in Table 2. Compared to subjects with normal ABI, subjects with abnormal ABI (low or borderline value) were more likely to be older, male, non-Hispanic white, and living alone. They also had a lower level of education, a smoking habit, a lower level of physical activity, a lower level of alanine aminotransferase, a lower level of aspartate aminotransferase, and diseases such as CVD, hypertension, and diabetes. BMI, economic status, triglyceride, HDL-cholesterol, fasting insulin, gamma glutamyltransferase, and fasting blood glucose were not significantly different between the three groups, but LDL-cholesterol and TC were. The baseline patient characteristics of the sample defined by USFLI are shown in supplementary Table 1.

**Table 2:** Characteristics of study population defined by hepatic steatosis index

	1.1 < ABI (*n* = 750)	0.9 < ABI ≤ 1.1 (*n* = 683)	ABI ≤ 0.9 (*n* = 107)	*P*-value
Age (years)	53.49 ± 0.42	56.24 ± 0.54	62.75 ± 1.72	< 0.01
Gender (%)				< 0.01
Male	429 (59.22)	244 (33.70)	44 (37.73)	
Female	321 (40.78)	439 (66.30)	63 (62.27)	
Ethnicity (%)				< 0.01
Non-Hispanic white	407 (79.71)	307 (70.79)	60 (76.18)	
Non-Hispanic black	90 (6.37)	163 (14.36)	25 (15.53)	
Mexican American	203 (5.35)	171 (5.80)	16 (2.43)	
Others	50 (8.58)	42 (9.06)	6 (5.85)	
Education level (%)				< 0.01
Less than high school	222 (15.54)	252 (24.40)	45 (31.59)	
High school or equivalent	181 (29.17)	154 (25.76)	27 (25.26)	
College or above	347 (55.29)	277 (49.84)	35 (43.15)	
Marital status (%)				< 0.01
Married or living with partner	544 (76.17)	419 (66.37)	62 (63.12)	
Others	206 (23.83)	264 (33.63)	45 (36.88)	
Family income-to-poverty ratio (%)				0.07
≤ 1.0	83 (6.58)	96 (9.97)	17 (9.63)	
> 1.0	619 (88.12)	527 (82.48)	82 (82.55)	
Unknown	48 (5.30)	60 (7.55)	8 (7.82)	
Smoker (%)				< 0.01
Never	377 (48.82)	345 (48.16)	29 (28.29)	
Ever	266 (35.88)	225 (30.54)	56 (44.61)	
Current	107 (15.30)	113 (21.30)	22 (27.10)	
BMI (kg/m^2^)	31.72 ± 0.25	32.20 ± 0.23	32.33 ± 0.88	0.34
Waist circumference (cm)	106.76 ± 0.66	106.21 ± 0.60	108.10 ± 1.70	0.41
Hypertension (%)				< 0.01
Without	360 (51.84)	245 (42.87)	29 (32.94)	
With	390 (48.16)	438 (57.13)	78 (67.06)	
Diabetes (%)				< 0.01
Without	609 (81.78)	527 (80.71)	67 (65.59)	
With	141 (18.22)	156 (19.29)	40 (34.41)	
CVD (%)				< 0.01
Without	661 (89.67)	584 (87.32)	80 (75.22)	
With	89 (10.33)	99 (12.68)	27 (24.78)	
PA level (%)				< 0.01
Low	310 (34.75)	335 (44.19)	62 (52.00)	
Moderate	171 (24.18)	130 (21.32)	24 (24.86)	
High	269 (41.07)	218 (34.49)	21 (23.13)	
Total cholesterol (mg/dL)	206.45 ± 2.23	213.83 ± 1.88	202.40 ± 4.34	< 0.05
HDL-cholesterol (mg/dL)	48.81 ± 0.65	51.06 ± 0.72	49.15 ± 1.50	0.06
LDL-cholesterol (mg/dL)	126.83 ± 2.12	131.89 ± 1.65	120.57 ± 3.97	< 0.01
Triglyceride (mg/dL)	154.21 ± 3.17	154.40 ± 2.92	162.94 ± 10.50	0.72
Fasting glucose (mg/dL)	108.32 ± 1.30	109.37 ± 1.43	114.67 ± 3.85	0.26
Fasting insulin (pmol/L)	83.18 ± 3.06	85.19 ± 2.69	99.89 ± 9.67	0.29
ALT (IU/L)	30.28 ± 0.84	25.80 ± 0.52	22.14 ± 1.30	< 0.01
AST (IU/L)	25.61 ± 0.61	23.23 ± 0.36	21.94 ± 0.84	< 0.01
GGT (IU/L)	32.67 ± 1.29	31.99 ± 1.50	35.47 ± 6.28	0.80

Data in the table: continuous variables are expressed as the weighted mean ± standard error; categorical variables are expressed as unweighted frequencies (weighted percentages)

BMI: body mass index; CVD: cardiovascular disease; PA: physical activity; ALT: alanine aminotransferase; AST: aspartate aminotransferase; GGT: gamma-glutamyl transferase; HDL: high density lipoprotein; LDL: low density lipoprotein

Over the median 13.25-year follow-up period, 330 subjects died (72 died of CVD). Table 3 shows the relationships between ABI and mortality in NAFLD patients. After controlling for demographic variables and smoking status (model 1), decreasing ABI had no effect on all-cause mortality. Even after adjusting for physical activity, coexisting diseases (hypertension, diabetes, cardiovascular disease), and waist circumference within model 2, ABI was not related to all-cause mortality. Furthermore, ABI was still not significantly related to all-cause mortality after including the metabolic confounders (HDL-cholesterol, LDL-cholesterol, triglycerides, fasting blood glucose, and fasting insulin) in the multivariable model (model 3). We used ABI as a continuous variable for sensitivity analysis and converted it to per 0.1 ABI (to make hazard ratio not too small). Similarly, no associations between ABI and all-cause mortality were found in any of the three models. When all of the above analyses were performed on the NAFLD sample defined by USFLI, the results were largely consistent (supplementary Table 2).

**Table 3:** Multivariate hazard ratio for mortality based on the ABI among individuals with NAFLD defined by hepatic steatosis index

Mortality	Deaths No./participants	Model 1	*P*	Model 2	*P*	Model 3	*P*
All-cause							
1.1 < ABI ≤ 1.4	132/750	Reference	0.02†	Reference	0.02^†^	Reference	0.03^†^
0.9 < ABI ≤ 1.1	148/683	0.90(0.65–1.25)	0.53	0.87(0.63–1.20)	0.41	0.87(0.63–1.20)	0.40
ABI < 0.9	50/107	1.58(0.94–2.68)	0.09	1.45(0.87–2.43)	0.16	1.38(0.82–2.32)	0.23
Per 0.1 ABI	330/1540	0.90(0.80–1.01)	0.06	0.92(0.83–1.03)	0.14	0.93(0.84–1.04)	0.20
Cardiovascular							
1.1 < ABI ≤ 1.4	24/750	Reference	0.02^†^	Reference	0.03^†^	Reference	0.04^†^
0.9 < ABI ≤ 1.1	33/683	1.21 (0.61–2.39)	0.58	1.08 (0.53–2.19)	0.83	1.16 (0.57–2.36)	0.69
ABI < 0.9	15/107	3.09 (1.38–6.93)	< 0.01	2.35 (1.03–5.37)	0.04	2.42 (1.10–5.33)	0.03
Per 0.1 ABI	72/1540	0.74 (0.65–0.85)	< 0.01	0.81 (0.71–0.93)	< 0.01	0.82 (0.71–0.93)	< 0.01

The multivariate model 1 was adjusted for age, gender, ethnicity, education level, marital status, Family income-to-poverty ratio and smoking status

The multivariate model 2 was further adjusted for waist circumference, hypertension, diabetes, cardiovascular disease and physical activity on the basis of model 1

The multivariate model 3 was adjusted for HDL-cholesterol, LDL-cholesterol, triglyceride and fasting blood glucose and fasting insulin in addition to model 2

ABI was converted into per 0.1 ABI after an increase of 10 times

All multivariate models in this table were analyzed with appropriate sampling weights

ABI: ankle–brachial index; NAFLD: nonalcoholic fatty liver disease; HDL: high density lipoprotein; LDL: low density lipoprotein

^†^*P*-values were analyzed using the test of trend of odds

**Fig. 2:**
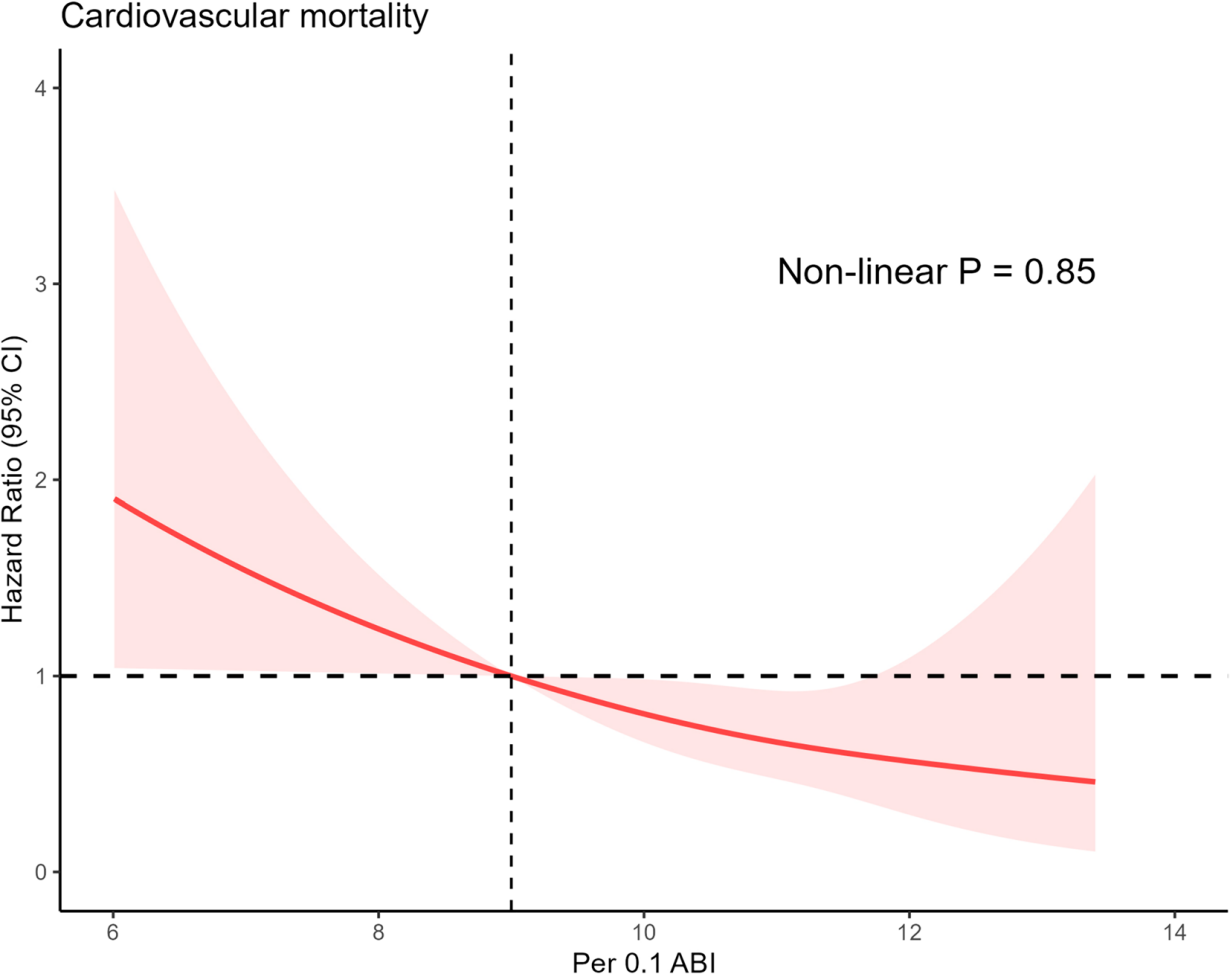
Association between per 0.1 ABI and cardiovascular mortality among patients with NAFLD defined by hepatic steatosis index. The red sold line represents the estimated hazard ratios, and the red shaded area represents the 95% confidence intervals. The restricted cubic spline function was adjusted for age, gender, ethnicity, education level, marital status, Family income-to-poverty ratio, smoking status, waist circumference, hypertension, diabetes, cardiovascular disease and physical activity, high density lipoprotein-cholesterol, low density lipoprotein-cholesterol, triglyceride, fasting blood glucose and fasting insulin. ABI was converted into per 0.1 ABI after an increase of 10 times. ABI: ankle–brachial index; NAFLD: nonalcoholic fatty liver disease

Then, in NAFLD cases, we conducted analyses of the relationships between ABI and CVM. Reduced ABI was associated with an increased risk of CVM in model 1 (HR: 3.09, 95% CI 1.38–6.93 for low ABI, P for trend = 0.02), and the results in model 2 were similar (HR: 2.35, 95% CI 1.03–5.37 for low ABI, P for trend = 0.03). After full adjustment, lower ABI was still associated with an increased risk of CVM (HR: 2.42, 95% CI 1.10–5.33, *P* for trend = 0.04). When ABI was used as a continuous variable in the sensitivity analysis, it was found to be negatively correlated with cardiovascular mortality in all models. Every 0.1 reduction in ABI increased the risk of cardiovascular mortality by 18% in the fully adjusted model (HR: 0.82, 95% CI 0.71–0.93). We used the restricted cubic spline function to confirm the linear relationship between ABI and cardiovascular mortality (Fig. 2). The results above were supported by a linear negative correlation between ABI and cardiovascular mortality (*P* for nonlinearity = 0.85). When the analyses were repeated using the NAFLD sample defined by USFLI, the results were nearly identical to those obtained using the NAFLD sample defined by HSI (Supplementary Table 2 and Supplementary Fig. 2).

Given that CVD is a risk factor for CVM and all-cause mortality, this study investigated the relationship of ABI level with mortality among NAFLD cases based on the presence of cardiovascular disease at baseline (Table 4). The interactions between ABI and CVD were also investigated (Table 4). In all three models, ABI was found to be unrelated to all-cause mortality in patients with NAFLD, regardless of the presence of CVD. In all multivariate models, there was no interaction between CVD and ABI. The outcome variable was then changed to cardiovascular mortality for analysis. In multivariable model 1, ABI was negatively correlated with cardiovascular mortality in individuals with CVD (HR: 0.73, 95% CI 0.61–0.86). After adjusting for additional risk factors, the correlations between ABI and cardiovascular mortality in models 2 and 3 remained consistent with those in model 1 (HR: 0.76, 95% CI 0.61–0.95 for model 2, HR: 0.68, 95% CI 0.51–0.91 for model 3). ABI was also negatively correlated with cardiovascular mortality in people who did not have CVD, and the correlations were identical in all three models (HR: 0.80, 95% CI 0.63–1.00 for model 1, HR: 0.83, 95% CI 0.72–0.95 for model 2, HR: 0.80, 95% CI 0.65–0.99 for model 3).

**Table 4:** Multivariate hazard ratio for mortality based on the ABI among individuals with NAFLD defined by hepatic steatosis index (stratified by the presence of baseline cardiovascular disease)

Mortality	Deaths No./participants	Model 1	*P*	Model 2	*P*	Model 3	*P*
All cause							
With CVD	88/215	0.92 (0.81–1.05)	0.21	0.95 (0.84–1.08)	0.42	0.91 (0.78–1.06)	0.21
Without CVD	242/1325	0.91 (0.79–1.04)	0.17	0.91 (0.79–1.04)	0.15	0.91 (0.79–1.04)	0.15
*P* for interaction		0.56		0.39		0.37	
Cardiovascular							
With CVD	30/215	0.73 (0.61–0.86)	< 0.01	0.76 (0.61–0.95)	0.02	0.68 (0.51–0.91)	0.01
Without CVD	42/1325	0.80 (0.63–1.00)	< 0.05	0.83(0.72–0.95)	< 0.01	0.80 (0.65–0.99)	0.04
*P* for interaction		0.90		0.58		0.90	

The independent variable used in this table is per 0.1 ABI, which is transformed from the increase of ABI by 10 times

The multivariate model 1 was adjusted for age, gender, ethnicity, education level, marital status, Family income-to-poverty ratio and smoking status

The multivariate model 2 was further adjusted for waist circumference, hypertension, diabetes and physical activity on the basis of model 1

The multivariate model 3 was adjusted for HDL-cholesterol, LDL-cholesterol, triglyceride and fasting blood glucose and fasting insulin in addition to model 2

All multivariate models in this table were analyzed with appropriate sampling weights

The interactions of CVD were tested

ABI: ankle–brachial index; NAFLD: nonalcoholic fatty liver disease; CVD: cardiovascular disease; HDL: high density lipoprotein; LDL: low density lipoprotein

We used NAFLD defined by the USFLI as a sample again for sensitivity analysis, and the results are shown in Supplementary Table 3. In Model 1, ABI was inversely correlated with all-cause mortality, irrespective of the presence or absence of CVD. After adjusting for further risk factors (Models 2 and 3), we discovered no links between ABI and all-cause mortality in NAFLD patients with or without CVD, which is consistent with observations made in NAFLD samples defined by HSI. When the relationship between ABI and cardiovascular mortality was examined, it was discovered that there was no correlation between ABI and CVM in CVD patients using the fully adjusted model (HR: 0.71, 95% CI 0.45–1.14 for model 3). However, after full adjustment, ABI was negatively correlated with cardiovascular mortality among individuals without CVD (HR: 0.61, 95% CI 0.48–0.77 for model 3), which was similar to the analysis based on HSI-defined samples. Furthermore, no interaction between CVD and ABI was found (*P* = 0.50 for model 3).

Given the association between diabetes and low ABI, we examined the relationship between ABI and CVM as well as all-cause mortality among NAFLD patients, stratified by the presence of diabetes at baseline (Table 5). In Model 1, ABI was inversely associated with all-cause mortality in NAFLD patients with diabetes (HR: 0.81, 95% CI 0.70–0.94), but there was no significant association between ABI and all-cause mortality in NAFLD patients without diabetes. After adjusting for additional risk factors, ABI was not significantly related to all-cause mortality in NAFLD patients, regardless of the presence or absence of diabetes (Models 2 and 3). In the analysis of CVM, ABI was inversely associated with cardiovascular mortality in NAFLD patients, irrespective of their diabetes status. Sensitivity analysis using the sample of USFLI-defined NAFLD showed results that were generally consistent with those defined by HSI (Supplementary Table 4).

**Table 5:** Multivariate hazard ratio for mortality based on the ABI among individuals with NAFLD defined by hepatic steatosis index (stratified by the presence of baseline diabetes)

Mortality	Deaths No./participants	Model 1	*P*	Model 2	*P*	Model 3	*P*
All cause							
With diabetes	115/337	0.81 (0.70–0.94)	0.01	0.88 (0.75–1.03)	0.10	0.89 (0.77–1.04)	0.15
Without diabetes	215/1203	0.97 (0.86–1.09)	0.59	0.97 (0.86–1.10)	0.69	0.98 (0.86–1.12)	0.81
*P* for interaction		0.13		0.28		0.36	
Cardiovascular							
With diabetes	40/337	0.67 (0.55–0.81)	< 0.01	0.72 (0.57–0.92)	0.01	0.72 (0.56–0.92)	0.01
Without diabetes	32/1203	0.87 (0.77–0.98)	0.03	0.90(0.73–0.97)	0.03	0.94 (0.69–0.98)	0.04
*P* for interaction		0.24		0.34		0.33	

The independent variable used in this table is per 0.1 ABI, which is transformed from the increase of ABI by 10 times

The multivariate model 1 was adjusted for age, gender, ethnicity, education level, marital status, Family income-to-poverty ratio and smoking status

The multivariate model 2 was further adjusted for waist circumference, hypertension, CVD and physical activity on the basis of model 1

The multivariate model 3 was adjusted for HDL-cholesterol, LDL-cholesterol, triglyceride and fasting blood glucose and fasting insulin in addition to model 2

All multivariate models in this table were analyzed with appropriate sampling weights

The interactions of diabetes were tested

ABI: ankle–brachial index; NAFLD: nonalcoholic fatty liver disease; CVD: cardiovascular disease; HDL: high density lipoprotein; LDL: low density lipoprotein

## Discussion

Our study provides novel insights into relationship between the ABI and mortality among patients with NAFLD, a patient group hitherto less studied in this context. We discovered a pronounced negative linear correlation between ABI and CVM within this cohort, a trend that persists even in the absence of CVD. Contradictory findings were observed regarding the relationship between low ABI and CVM among NAFLD patients with existing CVD. ABI was inversely related to cardiovascular mortality among NAFLD patients, regardless of diabetes status. Moreover, low ABI was not associated with all-cause mortality in patients with NAFLD.

NAFLD patients often carry a high metabolic burden and frequently present with multiple risk factors for CVD [6, 28]. CVD is currently the leading cause of death among patients with NAFLD. Although the ankle–brachial index (ABI) was initially developed to detect lower extremity arterial occlusions, any such occlusion usually indicates the presence of systemic atherosclerosis, a precursor to cardiovascular disease [7, 29, 30]. Low ABI was predictive of an increased risk of CVM in a study involving 5748 participants [31], supporting the observation in our study that low ABI is associated with increased CVM risk. Importantly, our research extends this association to patients with NAFLD. ABI, in addition to indicating potential cardiovascular disease, is also associated with many traditional CVD risk factors [29, 30]. Therefore, we speculate that NAFLD patients with low ABI, even without previous CVD, face an increased future risk of developing CVD, which in turn elevates their risk of CVM.

We discovered a different relationship between ABI and CVM among NAFLD cases with CVD in samples defined by two different panels [16, 17]. As previously reported, CVD cases with low ABI are associated with an increased CVM risk [32–34], and NAFLD individuals are also associated with an increased CVM risk [3, 5, 6]. Therefore, it is reasonable to assume that NAFLD patients may have an increased CVM risk when they have CVD and a low ABI. This hypothesis aligns with findings in samples defined by the HSI but not in those defined by the USFLI, where no correlation was observed. The discrepancy may be attributed to the significantly smaller sample size for the CVD subgroup in the USFLI-defined samples, potentially introducing bias in the analysis. Although the correlation was not statistically significant in these cases, the observed hazard ratios suggest a trend consistent with the results from HSI-defined samples, warranting further investigation in larger cohorts.

Our analysis reveals that regardless of diabetes status, ABI is inversely associated with cardiovascular mortality in patients with NAFLD. This finding underscores the value of ABI as a predictive marker for cardiovascular mortality in NAFLD patients, even in the absence of diabetes. Low ABI is linked to conventional CVD risk factors [29, 30], and diabetes stands as a strong indicator of cardiovascular mortality [35]. Consequently, it is not surprising that individuals with NAFLD who also suffer from diabetes and low ABI are at a substantially heightened risk for CVM. Notably, while the risk increase is more pronounced in the diabetic cohort, a low ABI also signifies elevated risk of cardiovascular mortality in non-diabetic individuals. These results consistently highlight the importance of ABI as an indicator for assessing the risk of cardiovascular mortality in NAFLD patients, irrespective of their diabetic status.

Furthermore, our research distinguished the impact of low ABI on CVM from all-cause mortality among NAFLD patients. Given that the primary cause of death in NAFLD patients is CVD, and ABI serves as an indicator of cardiovascular risk, it is logical to surmise a correlation between ABI and CVM in NAFLD. However, the predictive value of ABI for all-cause mortality appears to be limited. While low ABI has been associated with all-cause mortality in certain populations in some studies, these findings are not universally representative [36, 37]. Our research did not confirm a significant link between ABI and all-cause mortality in the NAFLD cohort, a discrepancy that could be attributed to in the study populations. Additionally, causes of death in NAFLD patients extend beyond CVD to include liver-related and cancer-related fatalities [18]. Presently, there is no direct evidence to suggest an association between ABI and these causes of death. This lack of association might also explain why our study did not find a correlation between low ABI and all-cause mortality.

Interestingly, the study by Ciardullo et al. also utilized the NHANES database to explore the relationship between ABI and NAFLD mortality [38]. While their findings regarding CVM were consistent with ours, they contrasted with our results on all-cause mortality. This discrepancy could be attributed to the distinct diagnostic models for NAFLD, differences in covariates, and the differing methods used for group categorization. Ciardullo et al. used a single non-invasive model, the fatty liver index, to define NAFLD, whereas our study enhanced robustness by utilizing two different non-invasive models, HSI and USFLI [16, 17, 38, 39]. In survival analysis, the model we constructed included covariates that differed from theirs, with the most significant difference being our inclusion of LDL-cholesterol, a well-known and influential risk factor in CVM [40]. Additionally, our approach to ABI categorization was more detailed. We divided NAFLD patients into more refined groups based on ABI ranges and explored dose–response relationships to determine if there were any inflection points. Ciardullo et al. conducted their analysis on the overall NAFLD population, whereas our study additionally performed stratified analyses based on the presence of diabetes or CVD [38]. Our results independently linked low ABI with an increased risk of CVM in NAFLD patients without previous CVD or diabetes. Considering that CVD and diabetes are significant risk factors for CVM, the risks associated with CVM are often underestimated in patients without these conditions. Thus, our findings highlight the predictive value of ABI for CVM in NAFLD patients without pre-existing CVD or diabetes. Moreover, through dose–response curves, we further explored the relationship between ABI levels and CVM, confirming a linear association between ABI and CVM in NAFLD patients. While the study by Ciardullo et al. primarily focused on the relationship between peripheral arterial disease (indicated by ABI < 0.90) and mortality in NAFLD patients, our research not only considers ABI as a categorical variable, but also analyzes it as a linear variable to more comprehensively evaluate its potential value in stratifying CVM risk among NAFLD patients [38].

ABI offers several advantages as a tool for predicting cardiovascular risk. It can be measured quickly by properly trained professionals within primary healthcare facilities, and it is a non-invasive examination [7, 41]. Our research indicates that low ABI is associated with an increased risk of CVM in patients with NAFLD. Therefore, ABI screening can be utilized as a valuable tool within primary healthcare settings to identify NAFLD patients at high risk for CVM. This approach may facilitate early intervention, potentially improving health outcomes for this population group.

Our research has some advantages. The data in this study have been tracked for more than ten years, and the non-institutional complex sampling stratified design is used in this study, which can better represent the general population in the United States. Second, the collection of biochemical and questionnaire data was conducted by trained personnel in a standardized and homogeneous manner. Third, we performed sensitivity analysis and adjusted many potential covariates to make the results more credible. However, there are some limitations to this work that should be mentioned. Since the NAFLD diagnosis is based on a non-invasive model that has not been validated by histology, the accuracy of the NAFLD diagnosis was limited. These two models, on the other hand, have been validated by ultrasound in a large population and are reliable non-invasive models [16, 17]. Second, due to the limitation of data on liver-related causes of death, we are unable to assess the associations between low ABI and liver-related mortality. Third, due to the small sample size, we were unable to further stratify the analysis by the degree of ABI reduction and further investigate the relationship between ABI in different ranges and cardiovascular mortality.

In conclusion, low ABI is independently associated with an increased risk of cardiovascular mortality in individuals with NAFLD. This correlation remains significant even in the absence of pre-existing cardiovascular disease or diabetes. However, ABI is unrelated to all-cause mortality. Routine ABI screening in patients with NAFLD may help in early identification of individuals at high risk of cardiovascular mortality, potentially enabling earlier intervention for these individuals. Nevertheless, more evidence is required to support this approach.

### Supplementary Information

Supplementary Material 1. Supplementary Fig. 1. Flow diagram of participants defined by US fatty liver index in the study.

Supplementary Material 2. Supplementary Fig. 2. Association between per 0.1 ABI and cardiovascular mortality among patients with NAFLD defined by US fatty liver index. The red sold line represents the estimated hazard ratios, and the red shaded area represents the 95% confidence intervals. The restricted cubic spline function was adjusted for age, gender, ethnicity, education level, marital status, Family income-to-poverty ratio, smoking status, waist circumference, hypertension, diabetes, cardiovascular disease and physical activity, high density lipoprotein-cholesterol, low density lipoprotein-cholesterol, triglyceride, fasting blood glucose and fasting insulin. ABI was converted into per 0.1 ABI after an increase of 10 times. ABI: ankle–brachial index; NAFLD, nonalcoholic fatty liver disease.

Supplementary Material 3. Supplementary Table 1. Characteristics of Study Population defined by US fatty liver index.

Supplementary Material 4. Supplementary Table 2. Multivariate Hazard Ratio for Mortality based on the ABI among Individuals with NAFLD defined by US Fatty Liver Index.

Supplementary Material 5. Supplementary Table 3. Multivariate Hazard Ratio for Mortality based on the ABI among Individuals with NAFLD defined by US Fatty Liver Index (Stratified by the presence of baseline cardiovascular disease).

Supplementary Material 6. Supplementary Table 4. Multivariate Hazard Ratio for Mortality based on the ABI among Individuals with NAFLD defined by US Fatty Liver Index (Stratified by the presence of baseline diabetes).

## Data Availability

Publicly available datasets were analyzed in this study. This data can be found here: https://www.cdc.gov/nchs/nhanes/index.htm.

## Abbreviations

ABI: Ankle–brachial index
NAFLD: Nonalcoholic fatty liver disease
HR: Hazard ratio
CI: Confidence interval
CVM: Cardiovascular mortality
SBP: Systolic blood pressure
CVD: Cardiovascular disease
NHANES: National Health and Nutrition Examination Survey
HSI: Hepatic steatosis index
USFLI: US fatty liver index
DBP: Diastolic blood pressure
ICD: International Classification of Diseases
MET-min: Metabolic equivalent-minute
MVPA: Moderate-to-vigorous physical activity
PA: Physical activity
NDI: National Death Index
BMI: Body mass index
TG: Triglyceride
HDL: High density lipoprotein
LDL: Low density lipoprotein
TC: Total cholesterol
