# Supplementary figures and images for: Low ankle–brachial index is associated with higher cardiovascular mortality in individuals with nonalcoholic fatty liver disease
Source: Eur J Med Res. 2024 May 9;29:276. doi: 10.1186/s40001-024-01878-5 (PMC11084075; doi:10.1186/s40001-024-01878-5)

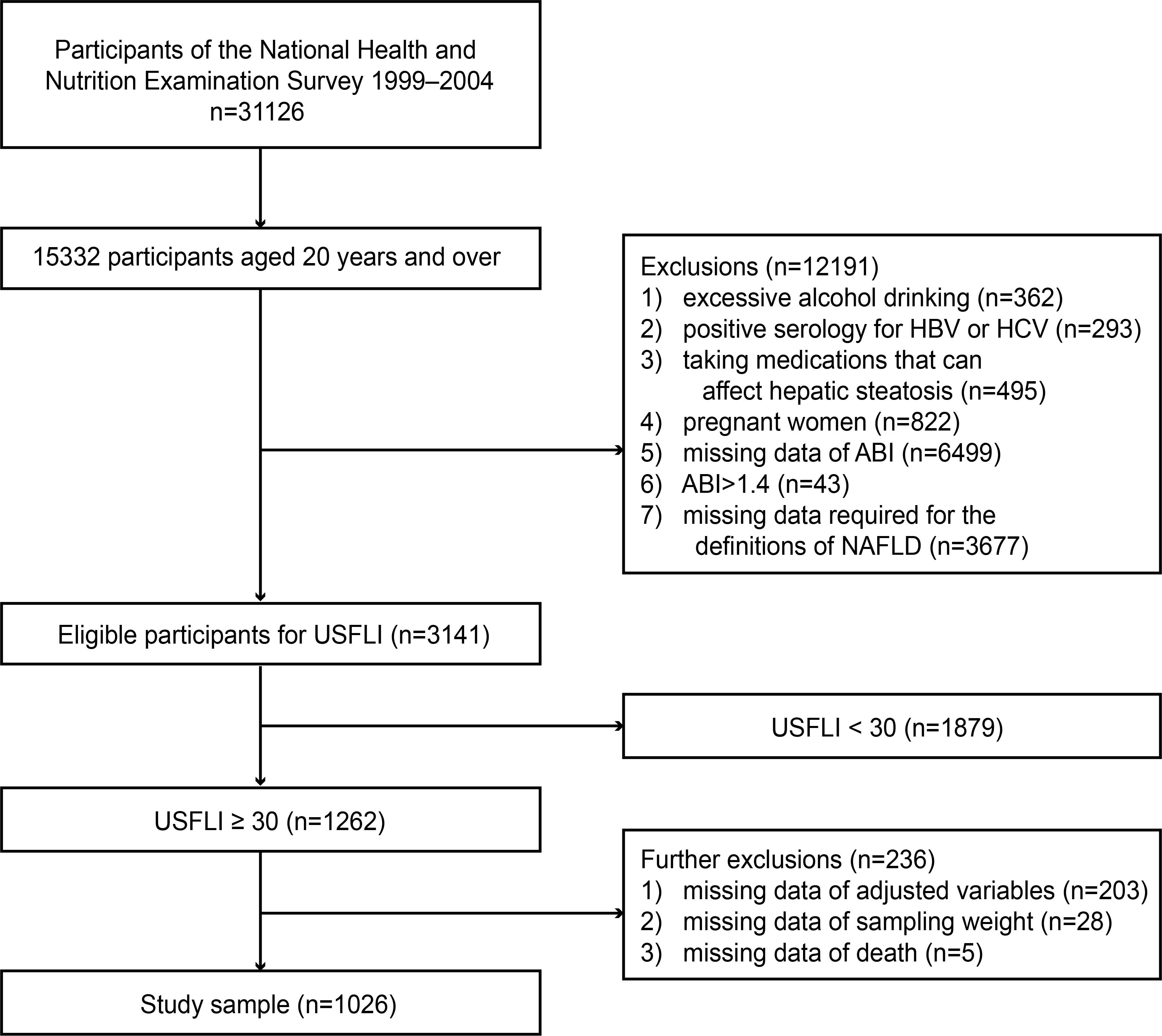

Supplement: Supplementary file 1 — Supplementary Material 1. Supplementary Fig. 1. Flow diagram of participants defined by US fatty liver index in the study. [file 40001_2024_1878_MOESM1_ESM.jpg]

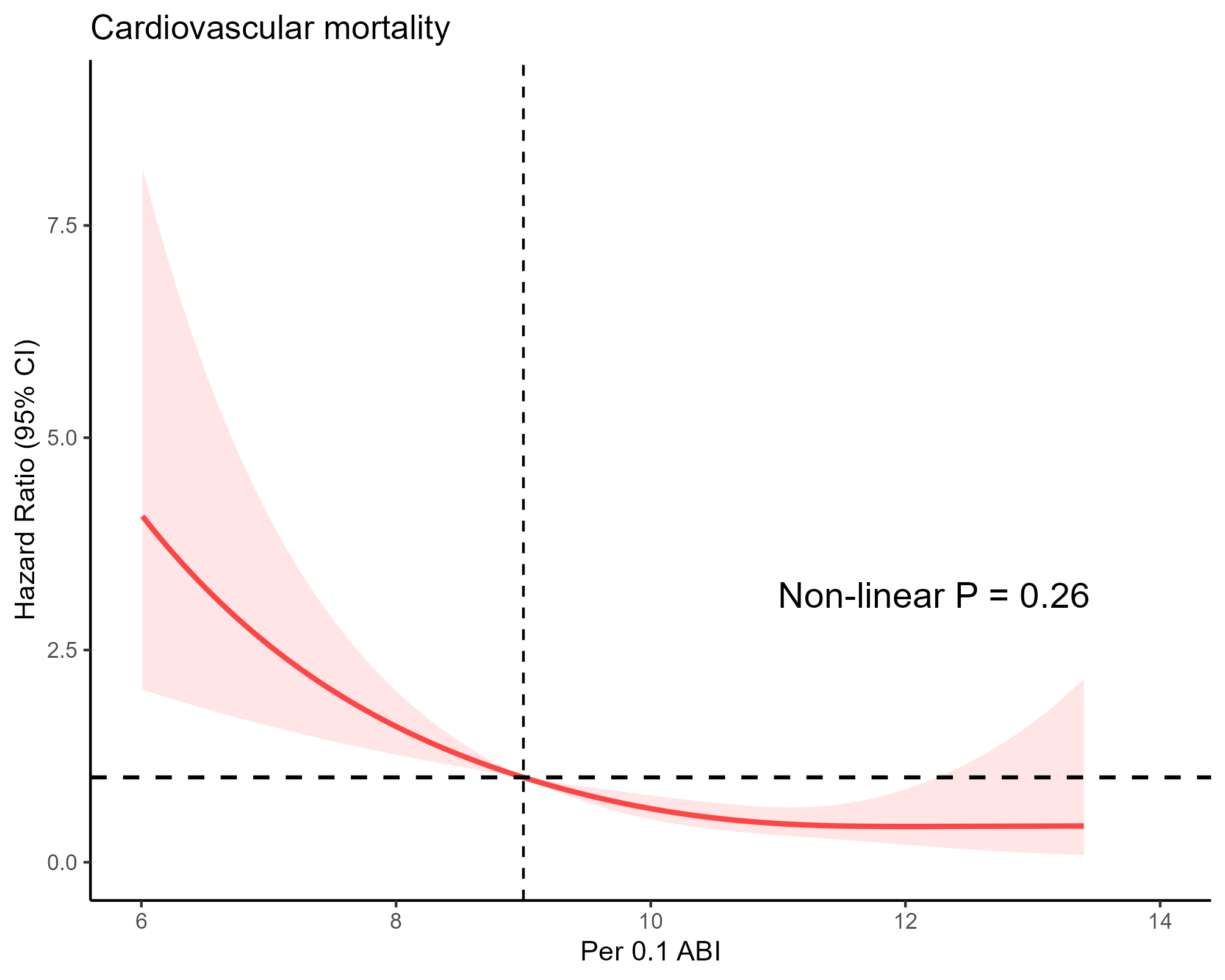

Supplement: Supplementary file 2 — Supplementary Material 2. Supplementary Fig. 2. Association between per 0.1 ABI and cardiovascular mortality among patients with NAFLD defined by US fatty liver index. The red sold line represents the estimated hazard ratios, and the red shaded area represents the 95% confidence intervals. The restricted cubic spline function was adjusted for age, gender, ethnicity, education level, marital status, Family income-to-poverty ratio, smoking status, waist circumference, hypertension, diabetes, cardiovascular disease and physical activity, high density lipoprotein-cholesterol, low density lipoprotein-cholesterol, triglyceride, fasting blood glucose and fasting insulin. ABI was converted into per 0.1 ABI after an increase of 10 times. ABI: ankle–brachial index; NAFLD, nonalcoholic fatty liver disease. [file 40001_2024_1878_MOESM2_ESM.jpg]
