# Supplementary material for: Low ankle–brachial index is associated with higher cardiovascular mortality in individuals with nonalcoholic fatty liver disease
Source: Eur J Med Res. 2024 May 9;29:276. doi: 10.1186/s40001-024-01878-5 (PMC11084075; doi:10.1186/s40001-024-01878-5)
Supplement: Supplementary file 3 — Supplementary Material 3. Supplementary Table 1. Characteristics of Study Population defined by US fatty liver index. [file 40001_2024_1878_MOESM3_ESM.docx]

**Supplementary Table 1.** Characteristics of Study Population defined by US fatty liver index.

|  | 1.1 < ABI  (n=501) | 0.9 < ABI ≤ 1.1  (n=438) | ABI ≤ 0.9  (n=87) | P-value |
| --- | --- | --- | --- | --- |
| Age (years) | 55.43 ± 0.51 | 58.99 ± 0.64 | 65.68 ± 2.10 | <0.01 |
| Gender (%) |  |  |  | <0.01 |
| Male | 329 (70.82) | 207 (46.01) | 47 (52.03) |  |
| Female | 172 (29.18) | 231 (53.99) | 40 (47.97) |  |
| Ethnicity |  |  |  | <0.01 |
| Non-Hispanic white | 277 (82.59) | 194 (72.80) | 48 (80.10) |  |
| Non-Hispanic black | 23 (2.42) | 54 (8.43) | 11 (8.74) |  |
| Mexican American | 171 (6.99) | 162 (8.55) | 23 (4.79) |  |
| Others | 30 ( 8.00) | 28 (10.23) | 5 ( 6.37) |  |
| Education level (%) |  |  |  | <0.01 |
| Less than high school | 171 (16.99) | 190 (29.76) | 39 (28.55) |  |
| High school or equivalent | 117 (28.27) | 94 (26.95) | 19 (23.69) |  |
| College or above | 213 (54.74) | 154 (43.29) | 29 (47.76) |  |
| Marital status (%) |  |  |  | <0.01 |
| Married or living with partner | 381 (79.81) | 271 (65.28) | 50 (61.52) |  |
| Others | 120 (20.19) | 167 (34.72) | 37 (38.48) |  |
| Family income-to-poverty ratio (%) |  |  |  | <0.05 |
| ≤ 1.0 | 56 (6.64) | 69 (12.76) | 17 (11.02) |  |
| > 1.0 | 413 (88.20) | 334 (79.86) | 62 (82.60) |  |
| Unknown | 32 (5.16) | 35 (7.38) | 8 (6.38) |  |
| Smoker (%) |  |  |  | <0.05 |
| Never | 245 (46.97) | 198 (42.25) | 21 (25.92) |  |
| Ever | 191 (39.11) | 165 (35.56) | 48 (48.42) |  |
| Current | 65 (13.92) | 75 (22.20) | 18 (25.66) |  |
| BMI (kg/m^2^) | 32.34 ± 0.30 | 32.65 ± 0.37 | 31.99 ± 1.25 | 0.65 |
| Waist circumference (cm) | 110.58 ± 0.76 | 109.76 ± 1.01 | 109.69 ± 1.64 | 0.75 |
| Hypertension (%) |  |  |  | 0.16 |
| Without | 207 (40.86) | 133 (36.67) | 24 (28.11) |  |
| With | 294 (59.14) | 305 (63.33) | 63 (71.89) |  |
| Diabetes (%) |  |  |  | <0.05 |
| Without | 380 (74.79) | 313 (73.73) | 50 (58.93) |  |
| With | 121 (25.21) | 125 (26.27) | 37 (41.07) |  |
| CVD (%) |  |  |  | <0.01 |
| Without | 428 (87.08) | 357 (82.12) | 62 (67.24) |  |
| With | 73 (12.92) | 81 (17.88) | 25 (32.76) |  |
| PA level (%) |  |  |  | 0.07 |
| Low | 227 (37.39) | 217 (45.27) | 44 (44.40) |  |
| Moderate | 106 (23.91) | 80 (19.30) | 23 (32.35) |  |
| High | 168 (38.70) | 141 (35.43) | 20 (23.25) |  |
| Total cholesterol (mg/dL) | 206.83 ± 2.74 | 210.91 ± 2.82 | 200.54 ± 4.91 | 0.19 |
| HDL-cholesterol (mg/dL) | 44.85 ± 0.60 | 47.43 ± 0.78 | 48.36 ± 1.92 | <0.01 |
| LDL-cholesterol (mg/dL) | 127.45 ± 2.65 | 128.66 ± 2.49 | 118.55 ± 4.84 | 0.14 |
| Triglyceride (mg/dL) | 172.91 ± 3.45 | 174.27 ± 4.98 | 168.14 ± 13.16 | 0.91 |
| Fasting glucose (mg/dL) | 116.44 ± 1.81 | 117.25 ± 2.38 | 120.57 ± 4.48 | 0.66 |
| Fasting insulin (pmol/L) | 106.74 ± 4.22 | 114.16 ± 3.73 | 126.28 ± 15.54 | 0.22 |
| ALT (IU/L) | 34.29 ± 1.27 | 29.41 ± 1.03 | 26.72 ± 2.82 | <0.01 |
| AST (IU/L) | 28.40 ± 1.05 | 25.83 ± 0.79 | 25.44 ± 1.84 | 0.12 |
| GGT (IU/L) | 39.98 ± 1.80 | 47.45 ± 3.98 | 50.23 ± 10.48 | 0.17 |

Data in the table: continuous variables are expressed as the weighted mean ± standard error; categorical variables are expressed as unweighted frequencies (weighted percentages).

BMI: body mass index; CVD: cardiovascular disease; PA: physical activity; ALT: alanine aminotransferase; AST: aspartate aminotransferase; GGT: gamma-glutamyl transferase; HDL: high density lipoprotein; LDL: low density lipoprotein.
