# Supplementary material for: Low ankle–brachial index is associated with higher cardiovascular mortality in individuals with nonalcoholic fatty liver disease
Source: Eur J Med Res. 2024 May 9;29:276. doi: 10.1186/s40001-024-01878-5 (PMC11084075; doi:10.1186/s40001-024-01878-5)
Supplement: Supplementary file 4 — Supplementary Material 4. Supplementary Table 2. Multivariate Hazard Ratio for Mortality based on the ABI among Individuals with NAFLD defined by US Fatty Liver Index. [file 40001_2024_1878_MOESM4_ESM.docx]

**Supplementary Table 2.** Multivariate Hazards Ratio for Mortality based on the ABI among Individuals with NAFLD defined by US Fatty Liver Index.

| Mortality | Deaths No./ participants | Model 1 | P | Model 2 | P | Model 3 | P |
| --- | --- | --- | --- | --- | --- | --- | --- |
| All-cause |  |  |  |  |  |  |  |
| 1.1<ABI≤1.4 | 113/501 | Reference | 0.07† | Reference | 0.08† | Reference | 0.08† |
| 0.9<ABI≤1.1 | 125/483 | 1.17(0.86-1.61) | 0.31 | 1.11(0.82-1.52) | 0.50 | 1.12(0.83-1.53) | 0.46 |
| ABI<0.9 | 48/87 | 2.67(1.55-4.66) | <0.01 | 1.88(0.99-3.55) | 0.05 | 1.84(0.98-3.46) | 0.06 |
| Per 0.1 ABI | 286/1026 | 0.80(0.71-0.90) | <0.01. | 0.86(0.75-1.00) | 0.05 | 0.86(0.75-1.01) | 0.06 |
| Cardiovascular |  |  |  |  |  |  |  |
| 1.1<ABI≤1.4 | 21/501 | Reference | <0.01† | Reference | <0.01† | Reference | <0.01† |
| 0.9<ABI≤1.1 | 26/483 | 1.16(0.64-2.08) | 0.63 | 1.03(0.52-2.03) | 0.94 | 0.93(0.46-1.89) | 0.85 |
| ABI<0.9 | 18/87 | 6.38(2.74-14.84) | <0.01 | 3.71(1.30-10.63) | 0.01 | 3.50(1.34-9.13) | 0.01 |
| Per 0.1 ABI | 65/1026 | 0.64(0.53-0.76) | <0.01 | 0.67(0.53-0.85) | <0.01 | 0.68(0.54-0.85) | <0.01 |

The multivariate model 1 was adjusted for gender, ethnicity, education level, marital status, Family income-to-poverty ratio and smoking status.

The multivariate model 2 was further adjusted for body mass index, hypertension, diabetes, cardiovascular disease and physical activity on the basis of model 1.

The multivariate model 3 was adjusted for HDL-cholesterol, LDL-cholesterol and triglyceride in addition to model 2.

ABI was converted into per 0.1 ABI after an increase of 10 times.

All multivariate models in this table were analyzed with appropriate sampling weights.

Abbreviations: ABI: ankle-brachial index; NAFLD: nonalcoholic fatty liver disease; HDL: high density lipoprotein; LDL: low density lipoprotein.

†P-values were analyzed using the test of trend of odds.
