# Supplementary material for: Low ankle–brachial index is associated with higher cardiovascular mortality in individuals with nonalcoholic fatty liver disease
Source: Eur J Med Res. 2024 May 9;29:276. doi: 10.1186/s40001-024-01878-5 (PMC11084075; doi:10.1186/s40001-024-01878-5)
Supplement: Supplementary file 6 — Supplementary Material 6. Supplementary Table 4. Multivariate Hazard Ratio for Mortality based on the ABI among Individuals with NAFLD defined by US Fatty Liver Index (Stratified by the presence of baseline diabetes). [file 40001_2024_1878_MOESM6_ESM.docx]

**Supplementary Table 4.** Multivariate Hazards Ratio for Mortality based on the ABI among Individuals with NAFLD defined by US Fatty Liver Index (Stratified by the presence of baseline diabetes).

| Mortality | Deaths No./ participants | Model 1 | p | | Model 2 | p | Model 3 | p |
| --- | --- | --- | --- | --- | --- | --- | --- | --- |
| All cause |  |  |  | |  |  |  |  |
| With Diabetes | 108/283 | 0.80(0.66-0.97) | 0.02 | | 0.82(0.67-1.00) | 0.05 | 0.79(0.66-1.01) | 0.06 |
| Without Diabetes | 178/743 | 0.84(0.72-0.97) | 0.02 | | 0.87(0.76-1.01) | 0.06 | 0.88(0.76-1.01) | 0.07 |
| P for interaction |  | 0.74 | | 0.95 | | | 0.91 | |
| Cardiovascular |  |  |  | |  |  |  |  |
| With Diabetes | 35/283 | 0.69(0.50-0.96) | 0.03 | | 0.65(0.48-0.89) | <0.01 | 0.62(0.48-0.81) | <0.01 |
| Without Diabetes | 30/743 | 0.65(0.48-0.87) | <0.01 | | 0.74(0.52-0.98) | 0.03 | 0.78(0.56-0.99) | 0.04 |
| P for interaction |  | 0.77 | | 0.93 | | | 0.77 | |

The independent variable used in this table is per 0.1 ABI, which is transformed from the increase of ABI by 10 times.

The multivariate model 1 was adjusted for gender, ethnicity, education level, marital status, Family income-to-poverty ratio and smoking status.

The multivariate model 2 was further adjusted for body mass index, hypertension, CVD and physical activity on the basis of model 1.

The multivariate model 3 was adjusted for HDL-cholesterol, LDL-cholesterol and triglyceride in addition to model 2.

All multivariate models in this table were analyzed with appropriate sampling weights.

The interactions of diabetes were tested.

Abbreviations: ABI: ankle-brachial index; NAFLD: nonalcoholic fatty liver disease; CVD: cardiovascular disease; HDL: high density lipoprotein; LDL: low density lipoprotein.
